# Feasibility, reproducibility, and prognostic value of a fully automated measurement of left ventricular longitudinal strain in heart transplant recipients

**DOI:** 10.3389/fcvm.2025.1499306

**Published:** 2025-05-20

**Authors:** Yu Cai, Chun Wu, Shuangshuang Zhu, Yanting Zhang, Yuji Xie, Yuting Tan, Xiaojun Yan, Lei Huang, Yichan Zhang, Yiwei Zhang, Yuman Li, Yali Yang, Jing Wang, Zhenxing Sun, Li Zhang, Mingxing Xie

**Affiliations:** ^1^ Department of Ultrasound Medicine, Union Hospital, Tongji Medical College, Huazhong University of Science and Technology, Wuhan, China; ^2^ Clinical Research Center for Medical Imaging in Hubei Province, Wuhan, China; ^3^ Hubei Province Key Laboratory of Molecular Imaging, Wuhan, China

**Keywords:** echocardiography, left ventricular longitudinal strain, fully automated measurement, heart transplant, application value

## Abstract

**Aims:**

Left ventricular longitudinal strain (LVGLS) is a robust parameter for predicting adverse events in patients who have undergone a heart transplant (HTx). However, measuring LVGLS is time-consuming and operator-dependent. Thus, we investigated whether automated strain software for LVGLS measurement has feasibility, reproducibility, and prognostic value in patients who underwent an HTx.

**Methods:**

In total, 286 patients who had undergone heart transplants and comprehensive echocardiography were included. LVGLS was obtained from the same apical images by three different methods, namely, fully automated LVGLS measurement (Auto-Strain), semiautomated LVGLS measurement (automated with manual editing), and manual LVGLS measurement. Patients were followed up and the primary composite endpoint (defined as all-cause death and rejection) was recorded.

**Results:**

Fully automated measurements were feasible in 277 subjects (96.8%). Analysis times for the automated LVGLS (27.7 ± 2.8 s/patient) and the semiautomated LVGLS measurement methods (237.4 ± 41.0 s/patient) were shorter than for the manual LVGLS measurement method (440.4 ± 65 s/patient). The semiautomated LVGLS measurement method showed a stronger correlation with the manual LVGLS measurement method than the automated LVGLS measurement method (*r* = 0.854 vs. 0.654, *P* < 0.001), and there were smaller disagreements between the semiautomated LVGLS and manual LVGLS measurement methods [bias: 0.79, limits of agreement (LOA): 2.78] than between the automated LVGLS and manual LVGLS measurement methods (bias: 2.72, LOA: 3.98). During a median follow-up of 51 months (35.0–66.5 months), 35 patients experienced endpoint events. The automated LVGLS measurement method can detect abnormal systolic function and predict adverse events in patients who have undergone an HTx, while the detection and prediction ability of semiautomated the LVGLS measurement method was greater.

**Conclusions:**

Fully automated LVGLS measurement enables rapid and reproducible assessment of graft function in patients who have undergone an HTx. Furthermore, the automated LVGLS measurement method detected abnormal systolic function and predicted adverse events, while the semiautomated LVGLS measurement method performed better in these aspects.

## Introduction

The clinical outcomes in heart transplantation (HTx) have demonstrated substantial improvements in recent decades (1). However, adverse post-transplant events such as acute graft rejection, cardiac allograft vasculopathy (CAV), and graft dysfunction continue to pose significant threats to the survival rate of patients who have undergone an HTx (2, 3). Thus, routine graft function surveillance is essential.

Echocardiography is recommended for annual monitoring of the cardiac function of post-transplanted hearts, primarily through measuring left ventricular ejection fraction (LVEF). Nevertheless, the prognostic ability of LVEF is limited in patients who have undergone an HTx (4). In contrast, left ventricular global longitudinal strain (LVGLS) has been demonstrated to be a robust and sensitive parameter for detecting subclinical ventricular dysfunction and predicting adverse cardiac events via evaluating left ventricular deformation (5–7), which is dependent on the longitudinal contractility of myocardial fibers located in the endocardium that are particularly vulnerable to ischemic and inflammatory injury (8). In spite of these advantages, LVGLS has not been popularized in routine clinical settings as its measurement is time-consuming and operator expertise-dependent (9, 10).

Recently, fully automated strain analysis software has been developed to delineate LV endocardial borders and measure LVGLS automatically, potentially addressing the aforementioned challenges. Several studies have demonstrated the feasibility of automated LVGLS measurement in different cohorts (11, 12). However, little research has applied fully automated LVGLS analysis software in patients who have undergone an HTx in large cohorts to assess its feasibility. Therefore, the present study was conducted with dual objectives: first, to determine the technical feasibility and reproducibility of an automated LVGLS measurement method in HTx recipients; second, to establish its prognostic value in patients who have undergone an HTx.

## Methods

### Study population

We included 286 recipients of heart transplants with a post-transplant period >6 months who had undergone comprehensive echocardiography in our hospital from January 2015 to December 2019. They were followed until 31 December 2021. The primary composite endpoint included all-cause death and graft rejection (defined as treatment of antibody-mediated rejection or grade ≥2R acute cellular rejection). The exclusion criteria were inability to perform strain analysis or acquire interpretable images. The study was approved by the Union Hospital Tongji Medical College Ethics Committee and complied with the Declaration of Helsinki.

### Echocardiography

Transthoracic echocardiographic examinations were performed using a Philips Epic 7c ultrasound system equipped with an S5-1 transducer (Philips Medical Systems, Andover, MA). Standard echocardiography parameters were acquired according to the recommendation of the American Society of Echocardiography (13), including left atrial volume index (LA volume/body surface area) and LV systolic and diastolic function.

### Assessment of LVGLS by different methods

LVGLS was obtained from standard apical views (two-, three-, and four-chamber views) according to the recommendation of the American Society of Echocardiography. LVGLS was measured using three different methods from the same image.

**Figure 1:**
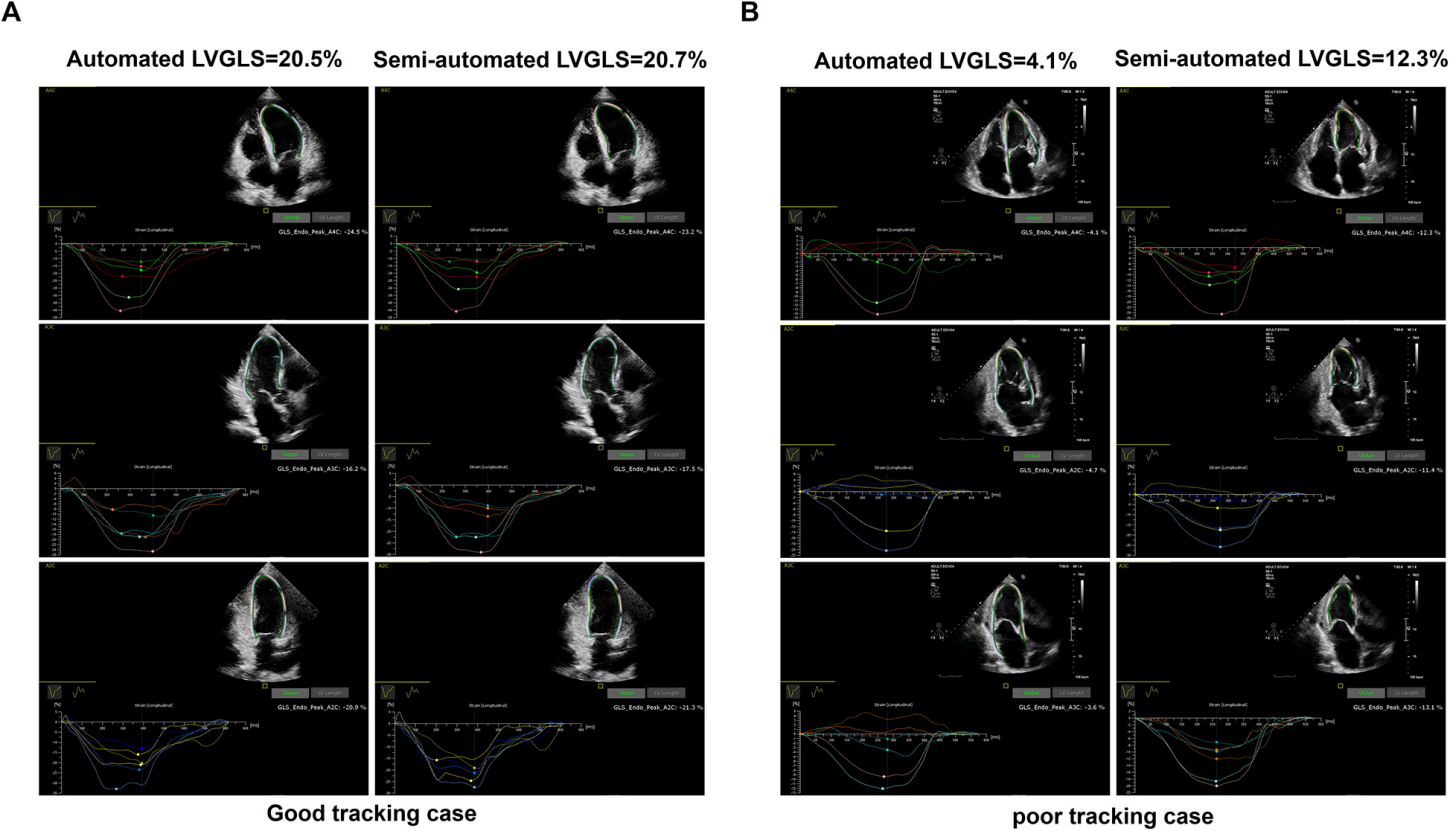
An example of good tracking (**A**) and poor tracking in LVGLS measurement (**B**) obtained from the automated and semiautomated assessments. The LVGLS values are absolute values.

(1) Automated LVGLS measurement: LVGLS was assessed via autoSTRAIN (TomTec-Arena, TomTec Imaging Systems, Unterschleissheim, Germany). After selecting the apical chamber view (the two-, three-, and four-chamber views) for analysis, the operator clicked the “auto-strain LV” button. Immediately, the endocardial border was automatically tracked and the LVGLS value was provided without manual correction. After the automatic measurement of LVGLS, the operator reviewed the tracking quality of the endocardial outline in each view. Images with good tracking quality were defined as having good tracking in more than four of six myocardial segments in each view (Figure 1).
(2) Semiautomated LVGLS measurement: After reviewing the tracking quality of the automated LVGLS measurement, the operator manually corrected the endocardial border if needed.
(3) Manual LVGLS measurement: LVGLS was obtained from the apical two-, three-, and four-chamber views via commercial software (2D Cardiac Performance Analysis 1.2, TOMTEC Imaging Systems GmbH, Unterschleissheim, Germany). The operator manually set three points in each view and then the software tracked the endocardial border automatically. Finally, the operator reviewed the tracking quality and made manual adjustments if needed.

### Time required to measure LVGLS

We randomly selected 50 patients to calculate the time required for each LVGLS measurement method. The time required to measure LVGLS was defined as the time from selecting three apical views from the menu to the results of LVGLS being acquired.

### Reproducibility

Intra- and inter-observer variability were evaluated through intra-class correlation coefficients (ICCs) and coefficients of variation (CVs) of the same images from the 50 randomly selected subjects. Intra-observer variability was evaluated by having the same investigator repeat the measurement 1 month later, and the investigator was blinded to the previous measurement. Inter-observer variability was analyzed between the first measurements of LVGLS conducted by two investigators blinded to each other's results. All the measurements were carried out by experienced investigators.

### Statistical analysis

Continuous variables were expressed as the mean ± SD or as the median (interquartile range). Categorical variables were presented as absolute values or percentages. Between-group differences in LVGLS measured by the three different methods were assessed using the Friedman test. Pearson's correlation analysis was used to assess the correlation among LVGLS measured using the three different methods. Bland–Altman analysis was applied to evaluate the agreement between two different methods via bias and limits of agreement (LOA). Receiver operating characteristic (ROC) curve analysis was used to evaluate the capability of the different methods to predict endpoint events, and areas under the ROC curves (AUCs) were calculated to compare the discriminating power of the three methods. The optimal cut-off value of LVGLS obtained from each method was also presented via ROC analysis. The manual LVGLS measurement method was taken as the gold standard to assess LV systolic function, and the capability of identifying abnormal LV systolic function was evaluated by ROC curves. The sensitivity, specificity, optimal cut-off values, and AUCs were calculated using ROC curve analysis. AUCs were used to compare the capability of different methods to identify abnormal LV systolic function. Time-to-event data were evaluated by Kaplan–Meier survival analyses. Intra-class correlation coefficients and ROC curves were compared using MedCalc Version 19.0.4 (MedCalc Software 7, Ostend, Belgium). Other statistical analyses were performed using SPSS version 22.0 (SPSS, Inc., Chicago, IL). A *p*-value <0.05 was considered to indicate statistical significance.

## Result

### Clinical and echocardiographic characteristics

The LVGLS values of 277 of the 286 patients (96.8%) were automatically measured, and the semiautomated and manual LVGLS measurements were also obtained from the 277 patients. Thus, this study involved 277 patients whose LVGLS was measured using the three different methods. Table 1 presents the baseline clinical and echocardiographic characteristics of the 277 subjects. The mean age of the recipients was 47.3 ± 12.5 years, and men constituted the majority of the study population (81.6%). According to previous studies (10), left atrial volume index (LAVI) (48.5 ± 18.7) and LV diastolic and systolic function [E/e’ ratio 7.1 ± 2.8; left ventricular peak systolic mitral annular velocity (LVS’) mean 8.8 ± 1.5; LVEF 61.7 ± 4.0%] were within the normal range.

**Table 1:** Baseline clinical and echocardiographic characteristics of heart transplant recipients.

Parameter	Value
Clinical characteristics	
Age (years)	47.3 ± 12.5
Time since transplantation (months)	51 (35–66.5)
Men, *n* (%)	226 (81.6%)
BMI (kg/m^2^)	23.1 ± 0.2
Systolic blood pressure (mm Hg)	119 ± 12
Diastolic blood pressure (mm Hg)	79 ± 10
Heart rate (beats/min)	91 ± 10
Comorbidities	
Diabetes, *n* (%)	153 (55.2%)
Hypertension, *n* (%)	132 (47.7%)
Echocardiographic characteristics	
Left atrial volume index (ml/m^2^)	48.5 ± 18.7
E velocity (cm/s)	81.8 ± 20.7
A velocity (cm/s)	47.5 ± 12.5
e’ (cm/s) (lateral)	12.4 ± 3.2
E/e’ (lateral)	7.1 ± 2.8
LVS’ mean (cm/s)	8.8 ± 1.5
LVEDV (ml)	88.9 ± 19.6
LVESV (ml)	34.1 ± 9.2
LVEF (%)	61.7 ± 4.0

e’, tissue Doppler mitral annulus early diastolic motion; E/e’, early mitral filling/tissue Doppler mitral annulus early diastolic motion; LVS’, left ventricular peak systolic mitral annular velocity; LVEDV, left ventricle end diastolic volume; LVESV, left ventricle end systolic volume; LVEF, left ventricle first-phase ejection fraction.

### Feasibility of automated measurement of LVGLS

**Figure 2:**
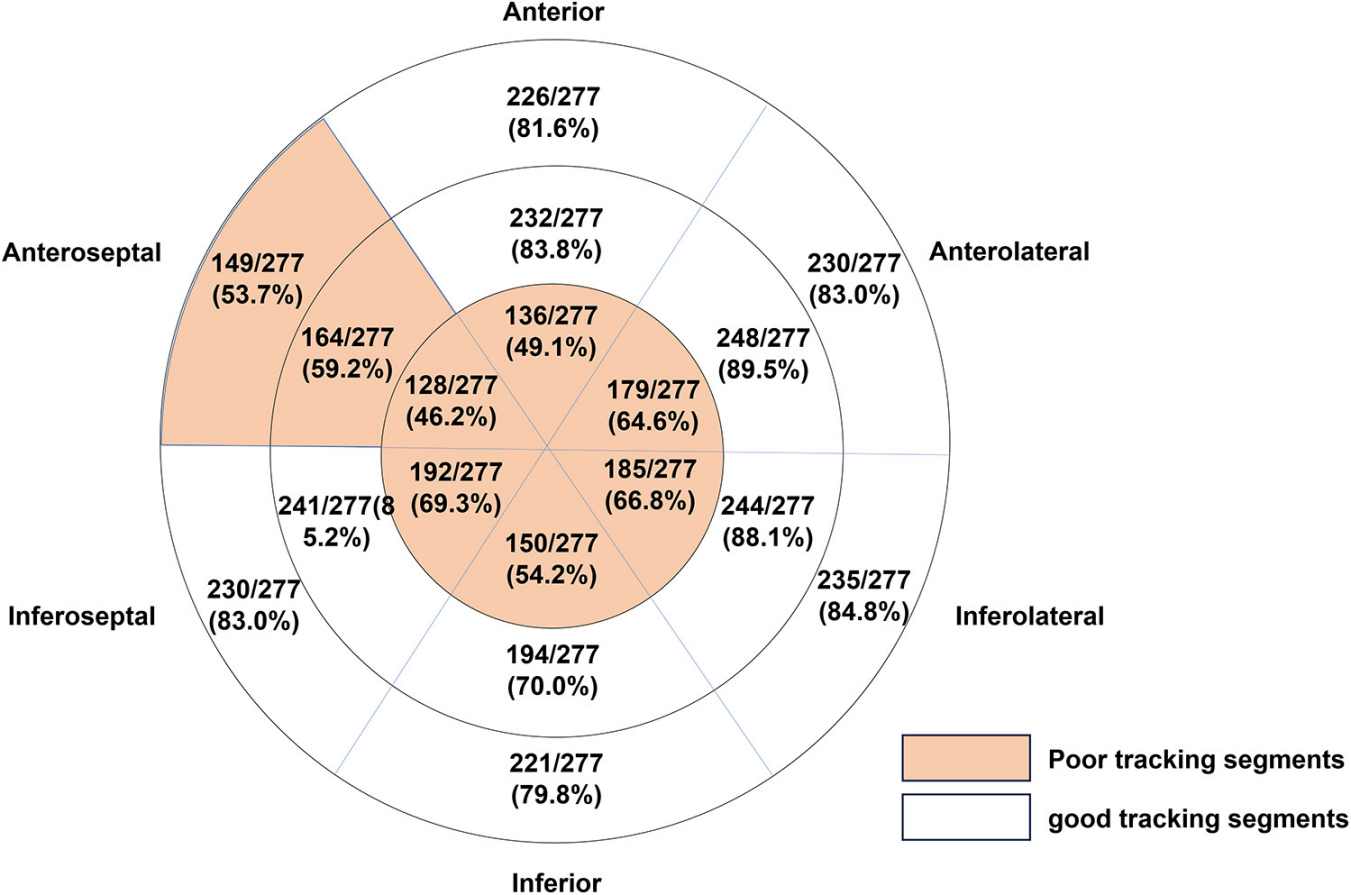
The tracking quality results in all segments are presented (*N* = 277). A good tracking segment was defined as the proportion of good tracking quality images being ≥70%.

The quality of the automated tracking was evaluated by an experienced investigator. The rate of good tracking in the apical four-chamber view (86.7%) was higher than that in the two-chamber (72.6%) and three-chamber views (68.2%), and the rate of good tracking in all views was 58.8%. The tracking quality results in all the segments are presented in Figure 2 and they revealed that the apical segments in all the views and the anteroseptal segments in the three-chamber view were challenging for the automated tracking and manual adjustments were needed.

### Comparison of LVGLS measured by different methods

**Figure 3:**
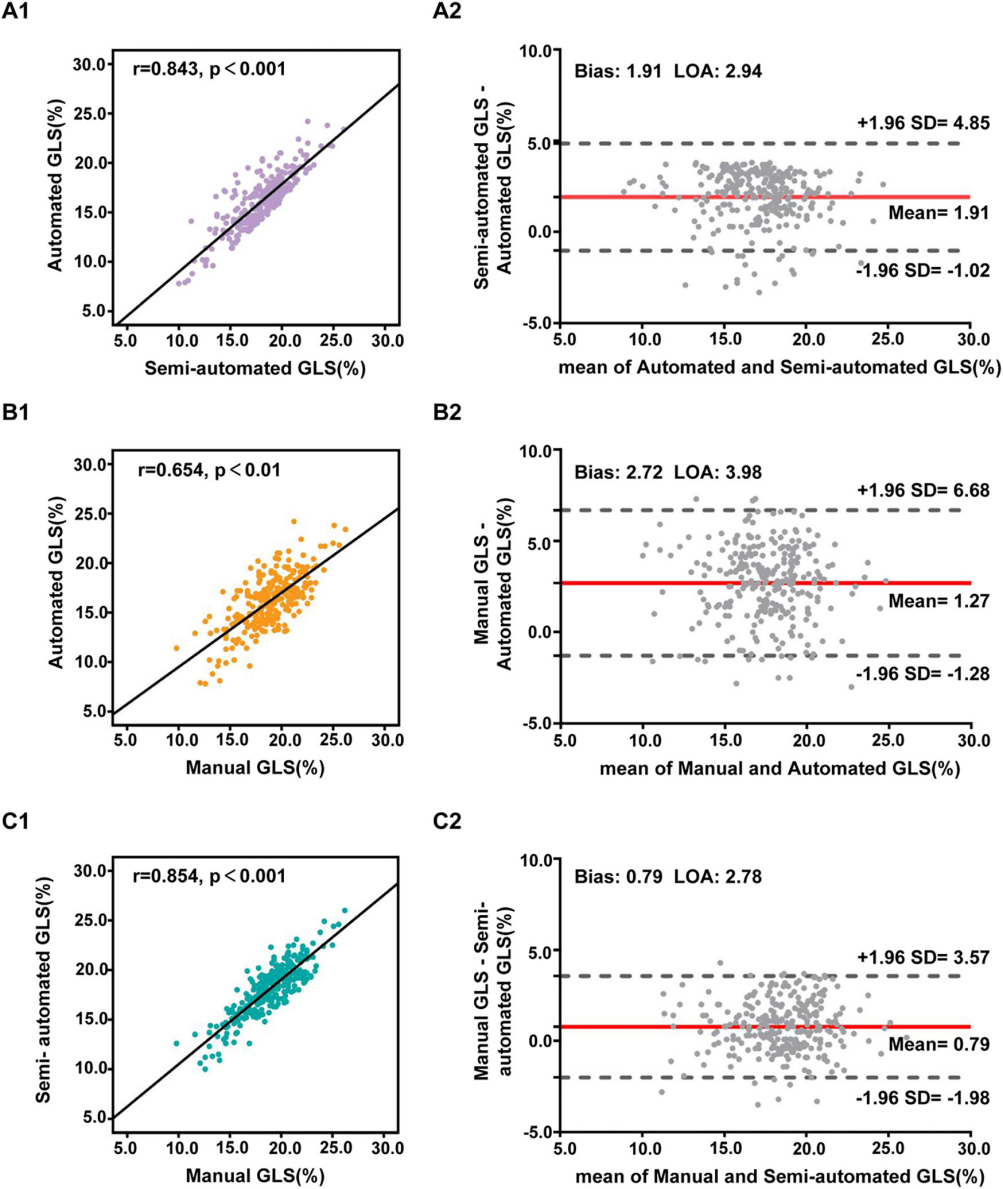
Pearson's correlation analysis (1) and Bland–Altman analyses (2) of the LVGLS measurements using different methods: automated and semiautomated assessment (**A**), automated and manual assessment (**B**), semiautomated and manual assessment (**C**). The LVGLS values are absolute values.

The mean values of the automated, semiautomated, and manual LVGLS measurement methods were 16.2 ± 2.7%, 18.1 ± 2.6%, and 18.9 ± 2.6%, respectively. The distribution of LVGLS obtained from the three different methods is presented as histograms in Supplementary Figure S1. The semiautomated and manual LVGLS measurement methods had similar mean values and spread, while the automated LVGLS measurement method showed a significant difference (*p* < 0.001). Furthermore, Pearson's correlation analysis revealed that the automated LVGLS measurement method was correlated with the manual LVGLS measurement method (*r* = 0.654, *p* < 0.01), while the correlation between the semiautomated and manual LVGLS measurement methods (*r* = 0.854, *p* < 0.001) was greater. The Bland–Altman analysis showed that the difference between the automated and manual LVGLS measurement methods was small (bias: 2.72, LOA: 3.98), but the divergence between the semiautomated and manual LVGLS measurement methods (bias: 0.79, LOA: 2.78) was smaller than that between the automated and manual LVGLS measurement methods (Figure 3 and Table 2).

**Table 2:** Comparison of LVGLS measured by the three different methods.

Method	*r*	*p*	Bias ± LOA
Automated vs. semiautomated	0.843	<0.001	1.91 ± 2.94
Automated vs. manual	0.654	<0.01	2.72 ± 3.98
Semiautomated vs. manual	0.854	<0.001	0.79 ± 2.78

### Capability of an automated LVGLS measurement method to detect and predict clinical adverse events

**Figure 4:**
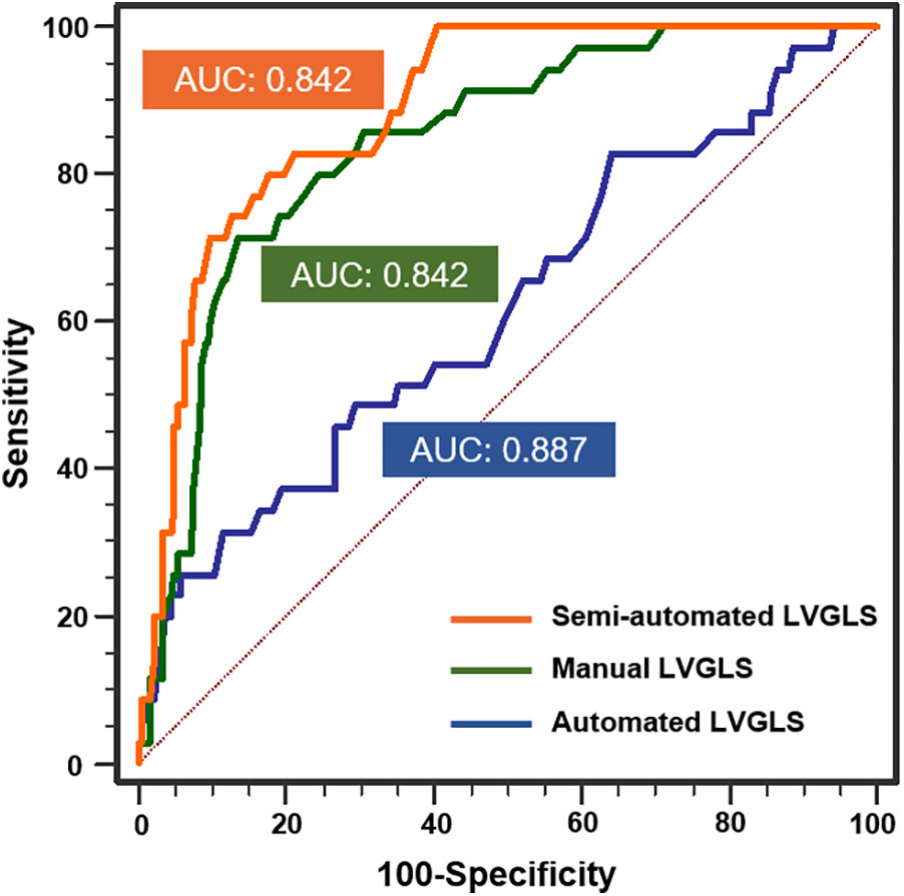
Receiver operating characteristic curves analyses of the automated, semiautomated, and manual LVGLS measurement methods for detecting adverse events. (*N* = 277, adverse events = 35).

**Figure 5:**
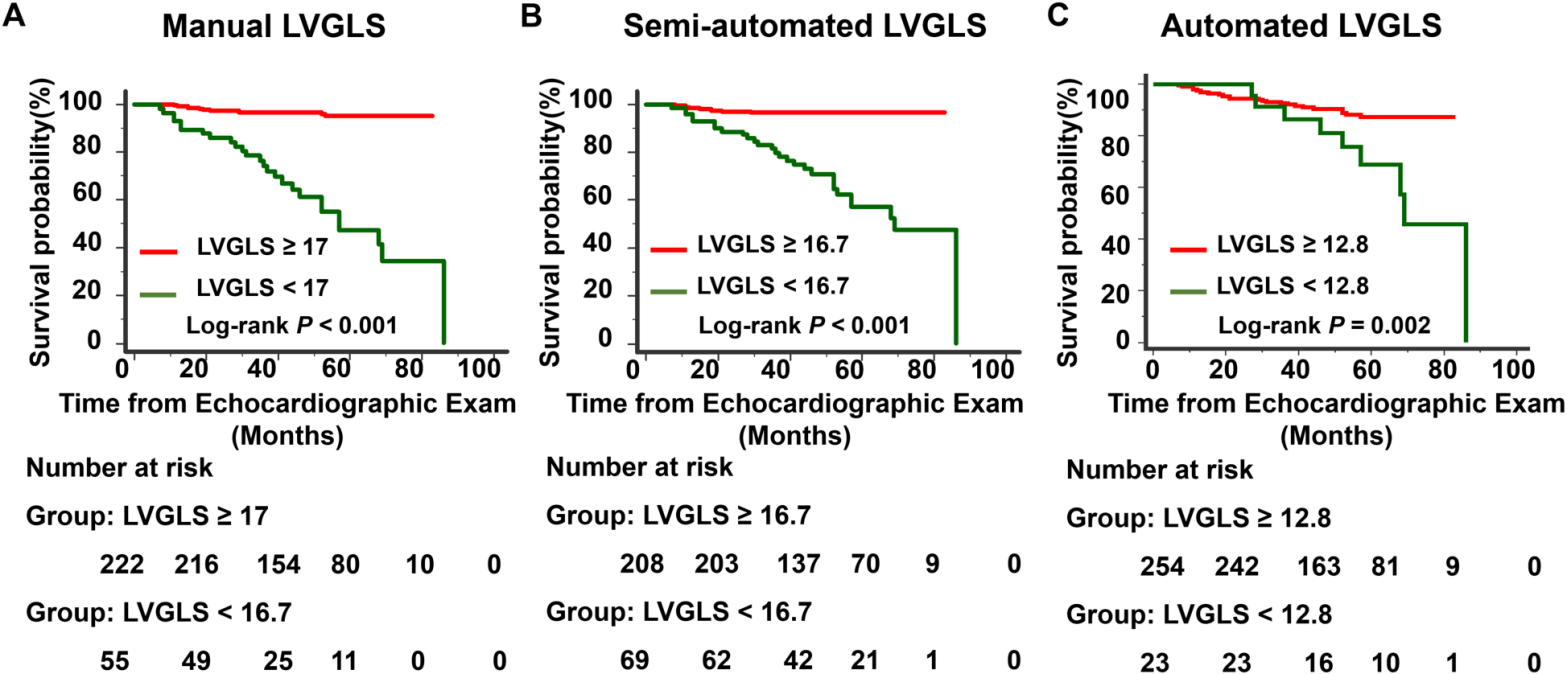
Kaplan–Meier event-free curves showing the association of LVGLS measured by three different methods with a higher risk of adverse events. The LVGLS values are absolute values. **(A)** Manual LVGLS, **(B)** Semi-automated LVGLS, **(C)** Automated LVGLS..

During a median follow-up of 51 months (35.0–66.5 months), 35 patients (12.6%) experienced endpoint events, including 20 deaths (10 from cardiac causes and 10 from non-cardiac causes) and 15 cases of rejection. The power of LVGLS obtained from three different methods to detect clinical adverse events was evaluated by ROC curve and AUC (Figure 4). All the LVGLS measurements obtained from the three methods could detect adverse events (*p* < 0.05 for all three). The AUC of the automated LVGLS measurement method was lower than that of the semiautomated and manual LVGLS measurement methods [0.617 (95% CI, 0557–0.674) vs. 0.887 (95% CI, 0.884–0.922), *p* < 0.001; 0.617 vs. 0.842 (95% CI, 0.794–0.883), *p* < 0.001]. The AUC of the semiautomated LVGLS measurement method was slightly higher than that of the manual LVGLS measurement method [0.887 (95% CI, 0.884–0.922) vs. 0.842 (95% CI, 0.794–0.883), *p* < 0.05]. The cut-off values of the automated, semiautomated, and manual LVGLS measurement methods were 12.8%, 16.7%, and 17%, respectively. The Kaplan–Meier survival curves stratified by cut-off values of LVGLS from the three methods were presented in Figure 5. The absolute values of the manual LVGLS, semiautomated LVGLS, and automated LVGLS measurement methods of <16.7%, <17%, and <12.8% were able to predict adverse clinical events, and the semiautomated LVGLS measurement method seemed to be the most effective.

### The automated LVGLS measurement method’s capability of detecting abnormal LV function

**Figure 6:**
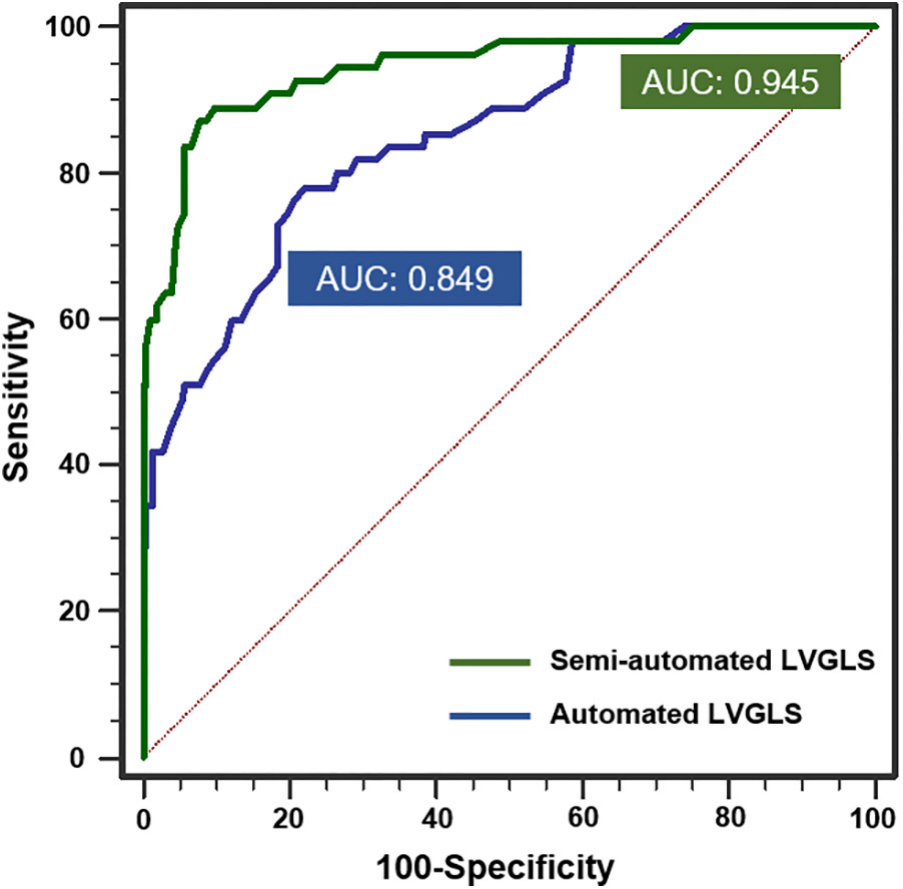
ROC curve analysis of the ability of the automated and semiautomated LVGLS measurement methods to detect abnormal systolic function when manual assessment was considered the gold standard.

The reference value of abnormal LVGLS for patients who had undergone an HTx has been studied, but no agreement has been reached (14, 15). Thus, we used the best cut-off value of the manual LVGLS measurement method (<17%) to detect adverse events as the gold standard for abnormal LV systolic function. The semiautomated and automated LVGLS measurement methods’ capability of detecting abnormal LVGLS was evaluated by an ROC curve analysis (Figure 6). The AUCs of the automated LVGLS and semiautomated LVGLS measurement methods for detecting abnormal LV systolic function were 0.849 (95% CI, 0.801–0.889) and 0.945 (95% CI, 0.911–0.969), respectively. According to the result, the automated LVGLS measurement method showed the ability to detect abnormal LVGLS, but the semiautomated LVGLS measurement method performed better (0.945 vs. 0.849, *p* < 0.001).

### Analysis of time required for LVGLS measurement

The median time required for the automated LVGLS, semiautomated LVGLS, and manual LVGLS measurement methods was 27.7 ± 2.8, 237.4 ± 41.0, and 440.4 ± 65 s/patient, respectively. The time required in the automated method was significantly shorter than that of the semiautomated and manual methods (*p* < 0.001 for both). The time required for the semiautomated LVGLS measurement method was significantly shorter than the time required for the manual LVGLS measurement method (*p* < 0.001).

### Reproducibility of LVGLS measurement

The intra- and inter-observer reproducibility are shown in Table 3. The ICCs of the intra-observer reproducibility for the automated LVGLS, semiautomated LVGLS, and manual LVGLS measurement methods were 1.0 (1.00–1.00), 0.95 (0.89–0.97), and 0.97 (0.91–0.99), respectively. The CVs of the intra-observer reproducibility for the automated LVGLS, semiautomated LVGLS, and manual LVGLS measurement methods were 0%, 2.01%, and 1.52%, respectively. The ICCs of inter-observer reproducibility for the automated LVGLS, semiautomated LVGLS, and manual LVGLS measurement methods were 1.0 (1.00–1.00), 0.92 (0.73–0.97), and 0.93 (0.87–0.96), respectively. The CVs of the inter-observer reproducibility for the automated LVGLS, semiautomated LVGLS, and manual LVGLS measurement methods were 0%, 2.47%, and 3.31%, respectively. Thus, the overall reproducibility of the three methods was good, while the automated LVGLS measurement method showed excellent reproducibility.

**Table 3:** Intra-observer and inter-observer variability in the LVGLS measurement methods.

Variable	CV (%)	ICC (95%)
Intra-observer		
Automated	0	1.00 (1.00–1.00)
Semiautomated	2.01	0.95 (0.89–0.97)
Manual	1.52	0.97 (0.91–0.99)
Inter-observer		
Automated	0	1.00 (1.00–1.00)
Semi-automated	2.47	0.92 (0.73–0.97)
Manual	3.31	0.93 (0.87–0.96)

CV, coefficients of variation; ICC, intra-class correlation coefficient; LVGLS, left ventricular longitudinal strain.

## Discussion

Based on available data, numerous studies have demonstrated the feasibility of automated GLS measurement (11, 12). However, few studies have specifically evaluated the feasibility of automated strain software in patients who have undergone a heart transplant. In our study, we confirmed the feasibility, reproducibility, and prognostic capability of an automated LVGLS measurement method in patients who had undergone an HTx. Furthermore, we provided reference values for LVGLS in clinically stable patients who had undergone an HTx. Several important findings were revealed: (1) the feasibility of the automated LVGLS measurement method was over 95%; (2) the fully automated LVGLS and semiautomated LVGLS measurement methods can be performed with low time requirement and excellent reproducibility; (3) the automated LVGLS measurement method had strong correlations with the semiautomated LVGLS and manual LVGLS measurement methods, while the semiautomated LVGLS measurement method was more correlated with the manual LVGLS measurement method; (4) the automated LVGLS measurement method possessed the capability to detect abnormal systolic function and predict clinical adverse events in medium and long term in patients who had undergone an HTx, but the semiautomated LVGLS measurement method showed better ability in these aspects.

Graft dysfunction is an important cause of late mortality after heart transplantation. Thus, it is vital to monitor the cardiac allograft function of patients who had undergone an HTx closely (16). The routine monitoring parameter used currently is LVEF obtained from echocardiography. Decreasing LVEF is associated with impaired myocardial function, but this parameter lacks sensitivity in detecting early-stage myocardial dysfunction (15). LVGLS has been proven to be a robust parameter and superior to LVEF in evaluating LV function accurately and reliably (17). Furthermore, the evaluation of LVGLS plays a vital role in diagnosing subclinical allograft dysfunction and predicting adverse clinical events. Thus, LVGLS measurement has been recommended to assess LV systolic function in echocardiographic guidelines (18). However, the accuracy and reliability of LVGLS measurement greatly rely on the experience of the operators, and measuring LVGLS is time-consuming (9, 10), which may hinder LVGLS from being widely used in clinical practice.

Recently, fully automated measurement of LVGLS has been developed and may provide a solution to these problems. A number of studies have shown that fully automated LVGLS measurement methods were useful in different cohorts, such as those with asymptomatic aortic stenosis and asymptomatic chronic aortic regurgitation (11, 12). However, few research studies have verified the feasibility, efficiency, and reproducibility of a fully automated LVGLS measurement method in patients who had undergone an HTx. In this study, we confirmed the feasibility, efficiency, and reproducibility of automated LVGLS and semiautomated LVGLS measurement methods, and furtherly compared the clinical implications of the three different methods. The feasibility of the automated LVGLS measurement method was over 95%, but it still had limitations. Its rate of good tracking in all chambers was only 58.8%, and over 40% of the automated LVGLS measurement data needed manual correction. These findings were consistent with previous studies (14). Some factors may be hindering its full potential: (1) poor image quality, which is associated with less agreement and greater bias of GLS measurement results (19), and (2) displacement and distorted anatomy of the transplanted heart, which make it difficult for automated analysis software to recognize the heart’s shape and endocardial border as automated analysis depends on knowledge-based identification of normal cardiac anatomy (20). The automated LVGLS measurement method showed greater reproducibility and efficiency than the semiautomated and manual LVGLS measurement methods. After taking prognostic value and measuring accuracy of the semiautomated LVGLS measurement method into consideration, it is possible to apply this automated method in daily clinical practice.

## Limitations

This study had several inherent limitations. First, this was a single-center study; a multi-center study will be needed to confirm our findings. Second, we performed automated assessment during sinus rhythm in all patients; further studies will be needed to clarify the usefulness of this assessment in patients with irregular heart rhythms. Third, although fully automated software packages for strain analysis from different vendors have much in common, they may use different algorithms, thus, the inter-vendor differences should not be neglected.

## Conclusion

A fully automated assessment of LVGLS possesses the power to efficiently monitor LV function and predict clinical adverse events in patients who have undergone an HTx, while the semiautomated approach performed better. Despite this, the fully automated method of measuring LVGLS provides the possibility of promoting LVGLS in clinical practice.

## Data Availability

The original contributions presented in the study are included in the article/Supplementary Material, further inquiries can be directed to the corresponding authors.

## Supplementary material

Supplementary Figure S1 The distributions of LVGLS measurements obtained from the three different methods are presented as histograms: the automated LVGLS measurement method (**A**), the semiautomated LVGLS measurement method (**B**), and the manual LVGLS measurement method (**C**) (*N* = 277).
