# Supplementary figures and images for: Feasibility, reproducibility, and prognostic value of a fully automated measurement of left ventricular longitudinal strain in heart transplant recipients
Source: Front Cardiovasc Med. 2025 May 20;12:1499306. doi: 10.3389/fcvm.2025.1499306 (PMC12129908; doi:10.3389/fcvm.2025.1499306)

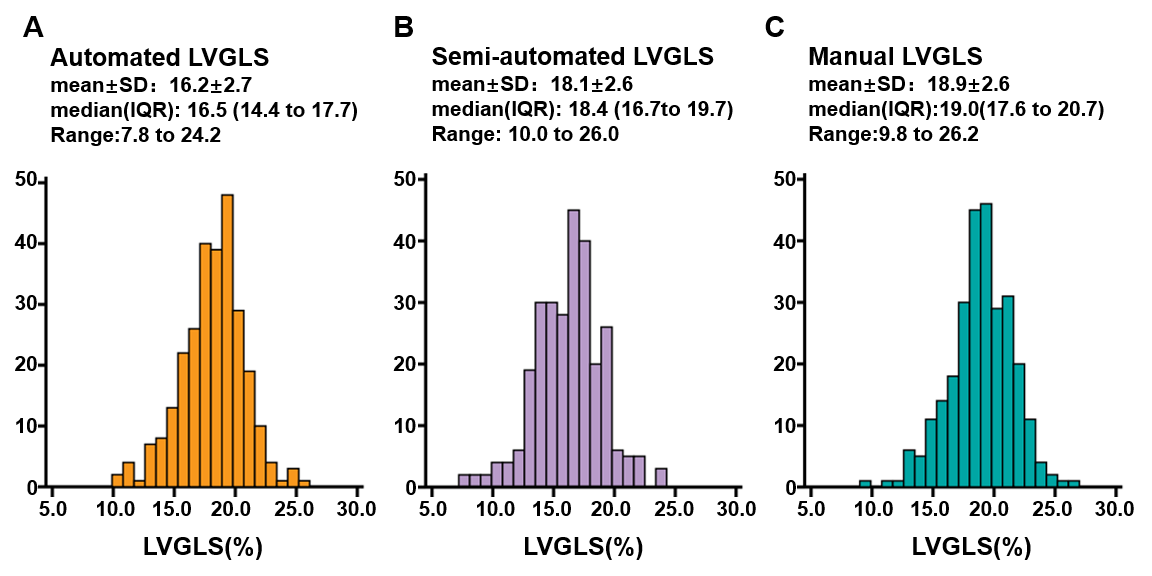

Supplement: Supplementary Figure S1 — The distributions of LVGLS measurements obtained from the three different methods are presented as histograms: the automated LVGLS measurement method (A), the semiautomated LVGLS measurement method (B), and the manual LVGLS measurement method (C) (N = 277). [file Image1.tif]
